# Supplementary material for: Breakdown of magnons in a strongly spin-orbital coupled magnet
Source: Nat Commun. 2017 Oct 27;8:1152. doi: 10.1038/s41467-017-01177-0 (PMC5658390; doi:10.1038/s41467-017-01177-0)
Supplement: Supplementary file 1 — Supplementary Information [file 41467_2017_1177_MOESM1_ESM.pdf]

SUPPLEMENTARY NOTE 1: PHASE DIAGRAMS OF THE EXTENDED $(J_1, K_1, \Gamma_1, J_3)$ MODEL

In this section, we compute the phase diagram for the extended $(J_1, K_1, \Gamma_1, J_3)$ spin Hamiltonian on the honeycomb lattice discussed in the main text, and given by:

$$\mathcal{H} = \sum_{\langle ij \rangle} J_1 \, \mathbf{S}_i \cdot \mathbf{S}_j + K_1 \, S_i^\gamma S_j^\gamma + \Gamma_1 \, (S_i^\alpha S_j^\beta + S_i^\beta S_j^\alpha) + \sum_{\langle\langle\langle ij \rangle\rangle\rangle} J_3 \, \mathbf{S}_i \cdot \mathbf{S}_j \tag{1}$$

where $\{\alpha, \beta, \gamma\} = \{y, z, x\}$ for the X bonds, $\{z, x, y\}$ for the Y bonds, and $\{x, y, z\}$ for the Z bonds shown in Fig. 1 of the main text. In order to capture the effect of local quantum fluctuations on the state energies, we computed second-order energy corrections to the classical ordered state energies, extending the method of Supplementary Ref. [1] to include finite Γ_1 and J_3. As discussed in Supplementary Refs. [1] and [2], an upper bound on the energies per site of the Kitaev spin-liquid states can be estimated from $\mathcal{E}_{\text{KIT}} = \pm\frac{3}{2}(J_1 + K_1)\langle S_i^\gamma S_j^\gamma \rangle$, where $\langle S_i^\gamma S_j^\gamma \rangle \approx 0.131$ is the analytical result for the first neighbour correlations at the pure $K_1 > 0$ or $K_1 < 0$ Kitaev points [3]. Comparison of $\mathcal{E}_{\text{KIT}}$ with the second-order corrected energies of the ordered states has been shown to reliably predict the position of the phase boundaries, which agree with the results of exact diagonalization (ED) [1, 2].

We show in Supplementary Figure 1a,b the phase diagram associated with Supplementary Eq. (1), parameterizing $J_1 = \cos\phi \sin\theta$, $K_1 = \sin\phi \sin\theta$, and $\Gamma_1 = \cos\theta$ as in Supplementary Ref. [4], with $J_3 = 0$ (Supplementary Figure 1a) and $J_3 > 0$ (Supplementary Figure 1b). The present results may be compared directly with the ED results of Supplementary Ref. [4] for $J_3 = 0$. The extended model of Supplementary Eq. (1) exhibits six ordered phases, which have been identified in various previous works [1–10]: FM = collinear ferromagnetic order, AFM = collinear Néel antiferromagnetic order, ST = collinear stripy order, ZZ = collinear zigzag order, 120 = noncollinear 120° order, and IC = incommensurate spiral order. In the extended model, the classical energies per site are given by:

$$\mathcal{E}_{\text{FM}} = \frac{1}{8}(3J_1 + K_1 - \Gamma_1 + 3J_3) \tag{2}$$

$$\mathcal{E}_{\text{AFM}} = -\frac{1}{8}(3J_1 + K_1 + 2\Gamma_1 + 3J_3) \tag{3}$$

$$\mathcal{E}_{120} = -\frac{1}{8}(K_1 + 2\Gamma_1) \tag{4}$$

$$\mathcal{E}_{\text{ST}} = \frac{1}{16}\left(-2J_1 + \Gamma_1 + 6J_3 - \sqrt{9\Gamma_1^2 - 4\Gamma_1 K_1 + 4K_1^2}\right) \tag{5}$$

$$\mathcal{E}_{\text{ZZ}} = \frac{1}{16}\left(2J_1 - \Gamma_1 - 6J_3 - \sqrt{9\Gamma_1^2 - 4\Gamma_1 K_1 + 4K_1^2}\right) \tag{6}$$

$$\mathcal{E}_{\text{IC}} = \frac{1}{2}\left(K_1 - \Gamma_1 - \sqrt{8\Gamma_1^2 + K_1^2}\right). \tag{7}$$

The full expressions with second-order corrections are lengthy, and therefore omitted here for brevity. However, the general trends are already apparent from Supplementary Eq. (2)-(7).

Of particular interest are the regions of stability of the zigzag order, observed experimentally in the honeycomb materials α-$RuCl_3$ and Na_2IrO_3, as well as the extent of the $K_1 > 0$

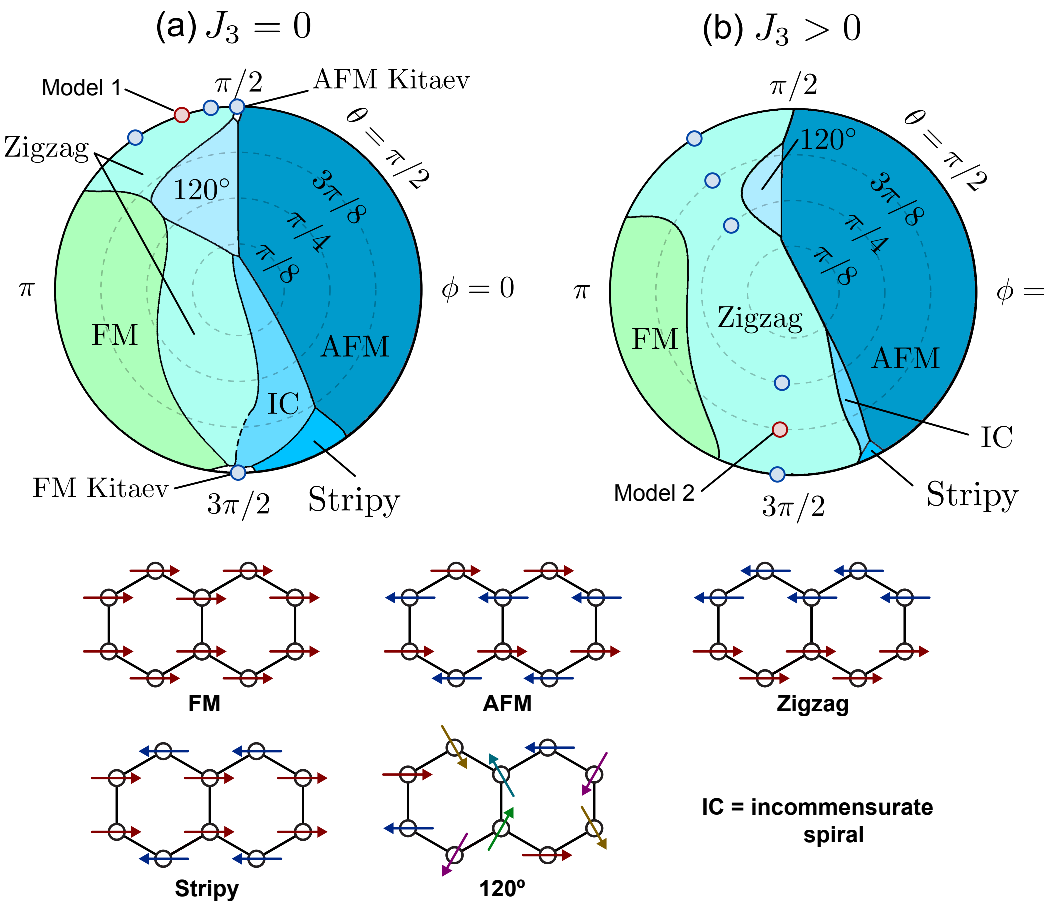

SUPPLEMENTARY FIGURE 1. **Phase diagrams for the extended model.** The phases were computed as described in the text. (a) With $J_3 = 0$. The dashed line between the incommensurate and zigzag phases indicates a region where $\mathcal{E}_{\rm ZZ} \approx \mathcal{E}_{\rm IC}$ and $\partial\mathcal{E}_{\rm ZZ}/\partial J \approx \partial\mathcal{E}_{\rm IC}/\partial J$, reducing the accuracy of the boundary line. Results here can be compared directly with the ED results of Supplementary Ref. [4]. (b) With a relatively small $J_3/\sqrt{J_1^2 + K_1^2 + \Gamma_1^2} = +0.088$ consistent with the magnitude in the studied models. Small circles indicate the considered parameters. For the incommensurate spiral state, we did not consider quantum modifications to the ordering wavevector. The models studied in Supplementary Note 5 are highlighted by blue and red points.

and $K_1 < 0$ Kitaev spin-liquids. For $J_3 = 0$, there are two zigzag regions, appearing at $(J_1 < 0, K_1 > 0, \Gamma_1 \approx 0)$, and $(J_1 \approx 0, K_1 < 0, \Gamma_1 > 0)$. The addition of a small finite J_3 uniquely stabilizes the zigzag and Néel states, linking the two zigzag regions, and suppressing the spin-liquid and other ordered phases. The existence of such J_3 coupling has been indicated for both α-$RuCl_3$ [8, 10] and Na_2IrO_3 [8, 9, 11]. This fact significantly complicates the identification of the magnetic interactions in these materials from investigations of the static properties alone, since the zigzag phase is stable over a very wide region of the phase diagram. The stability region for the $K_1 > 0$ and $K_1 < 0$ Kitaev spin-liquid states estimated from the second-order state energies is shown in Supplementary Table 1.

The relevant energy scale for considering the stability of the spin-liquid is the energy gap for spin-excitations at the pure Kitaev points, given by the two-flux gap $\Delta \approx 0.065|K_1|$

	$K_1 = +1$	$K_1 = -1$
$\Gamma_1 = 0, J_3 = 0:$	$-0.023 < J_1 < +0.025$	$-0.160 < J_1 < +0.095$
$J_1 = 0, J_3 = 0, \Gamma_1 > 0:$	$\Gamma_1 < +0.140$	$\Gamma_1 < +0.054$
$J_1 = 0, \Gamma_1 = 0, J_3 > 0:$	$J_3 < +0.041$	$J_3 < +0.053$

SUPPLEMENTARY TABLE 1. **Stability region for Kitaev spin-liquid states.** The regions were estimated from second-order state energies.

[12, 13]. Indeed, at the pure Kitaev points, the above estimates for the state energies suggest that the Kitaev state is stabilized with respect to adjacent magnetic orders by $0.072|K_1| \approx \Delta$ per site. In this sense, any perturbation on the scale of Δ has the potential to destabilize the spin-liquid. This explains why the Kitaev spin-liquid occupies a relatively small region of the phase diagram.

SUPPLEMENTARY NOTE 2: REVIEW OF *AB INITIO* STUDIES OF α-RuCl$_3$

In this section, we briefly review previous *ab initio* studies of the magnetic interactions in α-RuCl$_3$. As discussed by some of the present authors in Supplementary Ref. [8], estimation of such interactions from first principles calculations are complicated by several factors:

- The layered structure of α-RuCl$_3$ allows for significant stacking defects in the crystal structure, which have complicated structural solution. Very early studies indicated a trigonal space group $P3_112$ [14, 15], with $\angle$Ru-Cl-Ru $\sim 88°$. More recent detailed reanalysis of the structure suggested it to be monoclinic $C2/m$ [16, 17] at low temperatures, with $\angle$Ru-Cl-Ru $\sim 94°$. Furthermore, there is now increasing evidence that α-RuCl$_3$ exhibits a structural phase transition near $T \sim 150$ K [18, 19], which may be analogous to the $C2/m \rightarrow R\bar{3}$ transition of CrCl$_3$ [20].

  Given that the magnetic interactions are highly sensitive to the local geometry of the RuCl$_6$ octahedra, accurate estimation of their values has historically been complicated by the uncertainty in the crystal structure. This had led to a variety of reported interactions for α-RuCl$_3$, summarized in Supplementary Table 2.

- The low symmetry of the real crystal structures allows many independent terms to appear in the spin Hamiltonian. For example, up to 30 parameters are required to fully define the interactions up to third nearest neighbours [8]. As in the main text, most previous works have treated only a selection of such parameters, therefore assuming the interactions to be of higher symmetry than required by the crystal structure. This corresponds to an effective averaging of the interactions, which is likely to produce variations in the computed magnitudes of each parameter across different computational methods.

- For α-RuCl$_3$, the underlying energy scales (Hund's coupling, spin-orbit coupling, and crystal-field splitting, for example) are all of similar magnitude, which makes the computed interactions highly sensitive to fine details (such as structure, and choice

Structure	Method	sgn(K_1)	J_1/K_1	Γ_1/K_1	J_3/K_1	Ref.
$P3_112$ [14]	DFT+2OPT	$K_1 > 0$	-0.7	$+0.7$	$+0.02$	[21]
$P3_112$ [15]	DFT+ED	$K_1 > 0$	-0.7	$+1.1$	$+0.3$	[8]
$P3_112$ [14]	QC	$K_1 < 0$	$+0.4$	-0.8	–	[10]
$C2/m$ [22]	DFT+2OPT	$K_1 < 0$	$+0.1$	-0.5	–	[22]
$C2/m$ [16]	DFT+ED	$K_1 < 0$	$+0.25$	-1.0	-0.4	[8]
$C2/m$ [16]	QC	$K_1 < 0$	-0.1	-0.25	–	[10]
$C2/m$ [17]	DFT	$K_1 < 0$	$+0.15$	-0.35	-0.1	[23]
$C2/m$ [17]	DFT+2OPT	$K_1 < 0$	~ 0	-0.55	–	[24]

SUPPLEMENTARY TABLE 2. **Summary of previous interaction parameters for α-RuCl$_3$.** 2OPT = second order perturbation theory, ED = exact diagonalization, QC = quantum chemistry, and DFT = density functional theory. Since the magnitude of interactions may generally differ along the X, Y, and Z bonds, we present bond-averaged values.

of Coulomb parameters). This provides additional variations in computed interaction magnitudes appearing in the literature.

Despite these complications, estimates of the interaction parameters for α-RuCl$_3$ have broadly agreed across many different methods, when similar crystal structures are taken as input. A summary of previous *ab initio* calculations for such interactions is shown in Supplementary Table 2.

Ab initio studies based on the early $P3_112$ structures [14, 15] of α-RuCl$_3$ suggested antiferromagnetic $K_1 > 0$, with ferromagnetic $J_1 < 0$ and antiferromagnetic $\Gamma_1 > 0$ of similar magnitude [8, 21]. Microscopically, the positive K_1 arises from large direct hopping between Ru metal sites [4], which has a dramatic effect for the short Ru-Ru distances in the $P3_112$ structure. It should be emphasized that this hopping also generates large Γ_1 interactions, such that antiferromagnetic $K_1 > 0$ must always be accompanied by large Γ_1 in real materials. For this reason, pure (J_1, K_1) nnHK interactions suggested in Supplementary Ref. [25], based on spin-wave fitting, are inconsistent with $K_1 > 0$, from a microscopic perspective.

Later *ab initio* studies based on the (more accurate) $C2/m$ structures [16, 17] have instead suggested $K_1 < 0$, $J_1 \sim 0$, and $\Gamma_1 > 0$ [8, 10, 22–24]. For reasons suggested above, there has been a relatively large spread of computed values obtained from different computational methods. However, if we do not favour any particular method, we might expect an appropriate starting point for analysis to appear at the average values over all studies:

$$(J_1/K_1)_{\text{avg}} \sim +0.08 \quad , \quad (\Gamma_1/K_1)_{\text{avg}} \sim -0.5 \tag{8}$$

and $K_1 < 0$. As noted in the main text, excellent agreement between the ED and experimental neutron spectra is obtained for Model 2, with $(J_1/K_1) = +0.1$, and $(\Gamma_1/K_1) = -0.5$, which is completely consistent with the range of *ab initio* values appearing in the literature.

SUPPLEMENTARY NOTE 3: THREE-MAGNON COUPLINGS, DECAY KINEMATICS AND RATES, AND DYNAMICAL STRUCTURE FACTOR

Three-Magnon Coupling

In the main text we write: "a large decay rate is ensured by the following three conditions: large anisotropic interactions, deviation of the ordered moments away from the high-symmetry axes, and strong overlap of the one-magnon states with the multi-magnon continuum." Below we elaborate on these conditions for strong magnon decays.

The Hamiltonian for α-RuCl$_3$ is given by Supplementary Eq. (1). The diagonalization of the Hamiltonian requires rotation to the local reference frames of spins. Taking the zigzag ordering wavevector $\mathbf{Q}=$ Y, the ordered moment is expected to lie in the crystallographic ac-plane provided $\Gamma_1 > 0$, as for Models 1 and 2 of the main text, see also Supplementary Figure 2. This plane also contains the cubic z-axis. Thus, defining θ as the angle between the cubic z-axis and the ordered moment direction $\tilde{z}$, the spin operators can be rotated into the ordered coordinate frame via a rotation matrix:

$$\mathbf{R}_\theta = \begin{pmatrix} \cos^2\left(\frac{\theta}{2}\right) & -\sin^2\left(\frac{\theta}{2}\right) & \frac{1}{\sqrt{2}}\sin\theta \\ -\sin^2\left(\frac{\theta}{2}\right) & \cos^2\left(\frac{\theta}{2}\right) & \frac{1}{\sqrt{2}}\sin\theta \\ -\frac{1}{\sqrt{2}}\sin\theta & -\frac{1}{\sqrt{2}}\sin\theta & \cos\theta \end{pmatrix}, \tag{9}$$

where θ is obtained by a minimization of the classical energy and depends on K_1/Γ_1. Then the Hamiltonian is

$$\mathcal{H} = \sum_{\langle ij\rangle} \tilde{\mathbf{S}}_i \cdot \tilde{\mathbf{J}}_{ij} \cdot \tilde{\mathbf{S}}_j, \tag{10}$$

where $\tilde{\mathbf{J}}_{ij}$ is the bond-dependent exchange matrix in the local spin basis.

The coupling of the one- and two-magnon excitations is generated by the off-diagonal ("odd") terms that contain $\tilde{S}_i^z\tilde{S}_j^x$ and $\tilde{S}_i^z\tilde{S}_j^y$ in the local reference frame. Extracting such terms explicitly from the exchange matrix (10) gives

$$\mathcal{H}_{\rm odd} = \sum_{\langle ij\rangle} \left(J^{\rm odd}_{ij,x} S_i^x S_j^z + J^{\rm odd}_{ij,y} S_i^y S_j^z + i \leftrightarrow j\right), \tag{11}$$

For the zigzag structure with $\mathbf{Q}=$ Y, there are five distinct bonds with respect to the values of $J^{\rm odd}_{ij,x}$, $J^{\rm odd}_{ij,y}$. Thus, for the X and Y bonds, $J^{\rm odd}_{ij,x(y)} = \pm A_\pm$, with

$$A_\pm = \frac{\Gamma_1}{2}(\cos 2\theta \mp \cos\theta) + \frac{K_1}{2\sqrt{2}}(\cos\theta \pm 1)\sin\theta, \tag{12}$$

and for the Z bonds, $J^{\rm odd}_{ij,x(y)} = B$, where

$$B = \frac{1}{\sqrt{2}}(\Gamma_1 - K_1)\cos\theta\sin\theta. \tag{13}$$

Thus, magnon decay vertices scale as $\sim (A_\pm, B)$. It is immediately apparent that the exchange terms do not contribute to the three-magnon coupling because of the collinear spin arrangement in a zigzag structure [26].

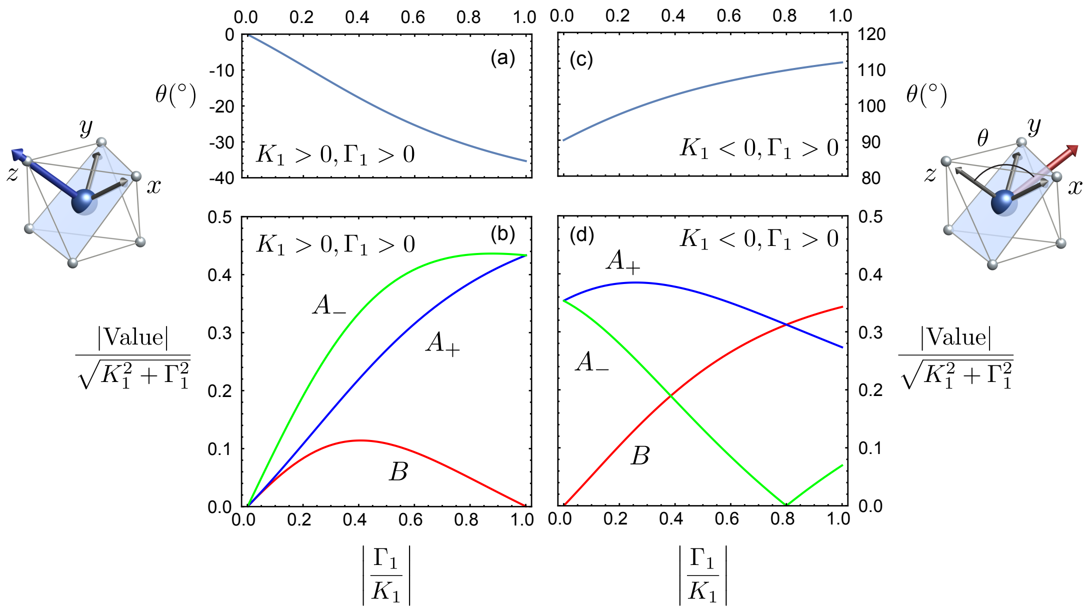

SUPPLEMENTARY FIGURE 2. **Off-diagonal exchange terms.** This figure shows the evolution of the direction of the ordered moment and of the off-diagonal ("odd") exchange terms (11) vs $|\Gamma_1/K_1|$. For (a) and (b), $K_1 > 0, \Gamma_1 > 0$, and for (c) and (d), $K_1 < 0, \Gamma_1 > 0$. In (b) and (d), terms contributing to the two-magnon decay vertex are shown. Their values are normalized to $\sqrt{K_1^2 + \Gamma_1^2}$, a rough magnitude of the single-magnon dispersion.

For the case of antiferromagnetic Kitaev coupling $K_1 > 0$, the ordered moments tend to align along the cubic z-axis, thus selecting $\theta \sim 0$ [27]. In Supplementary Figure 2a, we show the evolution of θ with $|\Gamma_1/K_1|$. For $\Gamma_1 = 0$, as in Model 1 of the main text, the ordered moment is exactly along the high-symmetry cubic z-axis ($\theta = 0$), and the off-diagonal $A_\pm$ and B terms vanish according to Supplementary Eqs. (12) and (13) above. As such, the decay of one-magnon excitations into the two-magnon continuum is forbidden in the zigzag phase for the pure Heisenberg and Kitaev interactions. In this case, all spin interactions in the local spin basis appear in the form of $\tilde{S}_i^x \tilde{S}_i^x$, $\tilde{S}_i^y \tilde{S}_i^y$, or $\tilde{S}_i^z \tilde{S}_i^z$, which contribute only to the terms of even order in magnon operators. As discussed in Supplementary Ref. [27], this ordered moment direction is stable with regard to small $\Gamma_1 > 0$, which shifts the moments to $\theta < 0$, with $\theta \propto |\Gamma_1/K_1|$. In this case, the average magnitude of the two magnon decay vertex is expected to scale linearly with the off-diagonal Γ_1 couplings for small Γ_1. That is $\Lambda \sim (A_\pm, B) \propto |\Gamma_1/K_1|$. The evolution of $(A_\pm, B)$ with $|\Gamma_1/K_1|$ is shown in Supplementary Figure 2b, normalized to the overall magnitude of interactions $\epsilon_{\mathbf{k},m} \sim \sqrt{K_1^2 + \Gamma_1^2}$, which sets the scale of the one-magnon bandwidth. One can see that the decay terms become significant compared to the one magnon dispersion for large Γ_1.

For the ferromagnetic Kitaev coupling $K_1 < 0$, and $\Gamma_1 > 0$ (as in Model 2 of the main text), the situation is somewhat different. Finite Γ_1 terms rotate the ordered moment away from the cubic z-axis by an angle $\theta \gtrsim 90°$ for any value of $|\Gamma_1/K_1| \gtrsim 0.05$ (see Supplementary Ref. [27]). Thus, the moments lie close to the cubic $\hat{x} + \hat{y}$ direction. In the rotated coordinate frame, both Γ_1 and Kitaev interactions contribute to the "odd" terms in (11), and thus induce two-magnon decays. Because of that, $A_\pm$ or B are always large and scale with the magnitude of K_1 and Γ_1, as shown in Supplementary Figure 2d. For that reason, in this

region of the phase diagram, the magnon decay vertex $\Lambda \sim (A_{\pm}, B) \sim (K_1, \Gamma_1)$ is always expected to be of the order of the one-magnon bandwidth.

The Holstein-Primakoff bosonization of (11) yields the three-boson Hamiltonian $\mathcal{H}_3$

$$\mathcal{H}_3 = \frac{1}{z} \sum_{\langle ij \rangle} \widetilde{J}_{ij}^{\mathrm{odd}} \left(a_i^\dagger a_j^\dagger a_j + \mathrm{H.c.} + i \leftrightarrow j \right), \tag{14}$$

where we extracted the coordination number $z = 3$. Here,

$$\widetilde{J}_{ij}^{\mathrm{odd}} \equiv 3\sqrt{\frac{S}{2}} \left(J_{ij,x}^{\mathrm{odd}} + i J_{ij,y}^{\mathrm{odd}} \right). \tag{15}$$

Using (12) and (13) for $S = 1/2$ and the parameters of Model 2, the three-magnon coupling for bonds X, Y and Z are

$$\left| \widetilde{J}_{X(Y)}^{\mathrm{odd}} \right| = 3.35\mathrm{meV}, \quad \left| \widetilde{J}_{Z}^{\mathrm{odd}} \right| = 2.78\mathrm{meV}. \tag{16}$$

The quantities in (16) can be referred to as the real-space three-magnon vertices. For Model 2, their strength relative to the full magnon bandwidth $W \approx 7$ meV is $\approx 0.4 - 0.5$. Such a strong anharmonic coupling is a precursor of strong magnon decays.

Decay Kinematics

The consideration above ensures that the amplitude of the anharmonic magnon-coupling terms is significant in case of Model 2 as well as for a wider part of the phase diagram. However, the effect of this coupling on the single-magnon states depends crucially on the availability of the two-magnon states for decays. A strong overlap of one-magnon states with the two-magnon continuum for the high-energy magnon branches is virtually guaranteed by the presence of the low-energy branches, as is exemplified favour of a significant overlap of such kind for all branches of the spectrum, including the lowest one. That argument, together with a strong three-magnon coupling, in turn guarantees substantial magnon line broadenings.

The situation of interest is illustrated in a sketch in Supplementary Figure 3. For a simple magnon branch with a Goldstone mode at $\mathbf{k} = 0$ one can see that the bottom of the two-magnon continuum is precisely degenerate with the one-magnon dispersion. Moreover, the density of the two-magnon states vanishes at the one-magnon energy, rendering decays impossible [26]. If a gap Δ is introduced in the magnon energy at $\mathbf{k} = 0$, the two-magnon continuum will still have a minimum at the same Γ point, but will be gapped with the energy 2Δ.

Next is the case of the gapless modes occurring at finite $\pm\mathbf{k}_0$, keeping an overall shape of the dispersion as simple as possible. It is obvious that now the two magnons with the total momentum $\mathbf{q} = 0$ have the bottom of their continuum at $\mathrm{Min}(\epsilon_{\mathbf{k}} + \epsilon_{-\mathbf{k}}) = 0$. Thus, the two-magnon continuum must be below the one magnon states in an extended vicinity of $\mathbf{q} = 0$. It is also obvious that this argument is robust against a finite gap at the (pseudo-) Goldstone point as the overlap of the magnon branch with the continuum survives for a finite Δ.

We mention that such a situation is not uncommon and describes almost exactly the case of a spiral antiferromagnet, which has an ordering vector at finite momentum, see

[26]. Another generic case are the spin-ladder and 2D valence-bond-like antiferromagnets having band minima at a finite **Q**. The decay kinematic conditions and magnon decays are well-documented for them, both theoretically and experimentally, see [26] and [28, 29] for a recent realization.

While, in general, one can formulate a complete list of checks for decay conditions for an arbitrary form of the magnon spectrum, see [26], the current consideration provides a clear and intuitive picture of why magnon decays must occur in Model 2 and also more broadly.

Decay Rates

Using standard diagrammatic rules with the decay terms, one can straightforwardly obtain magnon self-energies in the one-loop (Born) and on-shell approximations, $\Sigma_{\mu\mathbf{k}}(\omega) \to \Sigma_{\mu\mathbf{k}}(\varepsilon_{\mu\mathbf{k}})$, both strictly within the $1/S$ expansion [26]. This neglects such effects as more complicated spectrum renormalizations and spectral weight redistribution away from the quasiparticle pole. One can argue that the real part of the self-energy should be neglected altogether as the renormalization of magnon energies is already built in by the choice of the model parameters. In practice, the LSWT parameters are indeed often chosen to best fit the observed experimental and/or numerical bands.

Altogether, this leaves us with the only remaining and yet the most important and physically distinct effect of the anharmonic terms: magnon decays. Thus, the self-energy of the magnon branch μ is $\Sigma_{\mu\mathbf{k}}(\omega) \to -i\gamma^{\mu}_{\mathbf{k}}$, and the decay rate in the Born approximation is

$$\gamma^{\mu}_{\mathbf{k}} = \frac{\pi}{2}\left|\widetilde{J}^{\text{odd}}\right|^2 \sum_{\mathbf{q},\eta\nu} \left|\widetilde{\Phi}^{\eta\nu\mu}_{\mathbf{q},\mathbf{k}-\mathbf{q};\mathbf{k}}\right|^2 \delta(\varepsilon_{\mu\mathbf{k}} - \varepsilon_{\eta\mathbf{q}} - \varepsilon_{\nu\mathbf{k}-\mathbf{q}}). \tag{17}$$

We take $\widetilde{J}^{\text{odd}}$ as an average value from (16), and $\widetilde{\Phi}^{\eta\nu\mu}_{\mathbf{q},\mathbf{k}-\mathbf{q};\mathbf{k}}$ is a dimensionless function of eigenvectors from the diagonalization of the quadratic Hamiltonian.

The decay rate is related to a much simpler quantity, the on-shell two-magnon density of states (DOS),

$$D^{\mu}_{\mathbf{k}} = D_{\mathbf{k}}(\varepsilon_{\mu\mathbf{k}}) = \pi \sum_{\mathbf{q},\nu\eta} \delta\left(\varepsilon_{\mu\mathbf{k}} - \varepsilon_{\nu\mathbf{q}} - \varepsilon_{\eta\mathbf{k}-\mathbf{q}}\right). \tag{18}$$

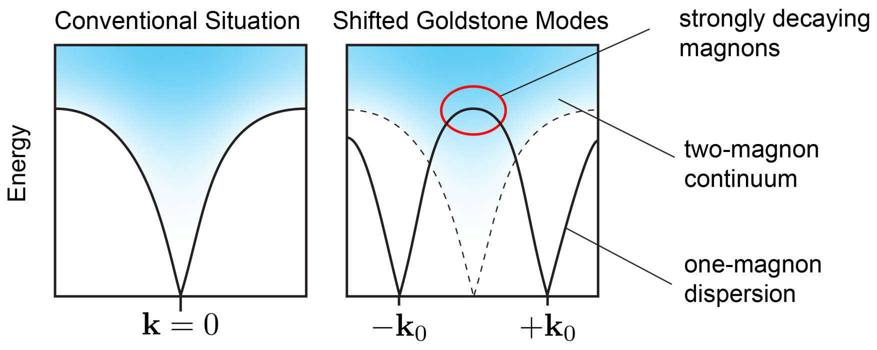

SUPPLEMENTARY FIGURE 3. **Kinematic conditions for two-magnon decay.** This sketch shows the kinematic conditions for two-magnon decay emphasizing the importance of the (pseudo-) Goldstone mode at a finite **Q**-value.

We propose to approximate the square of the dimensionless vertex $\big|\widetilde{\Phi}^{\eta\nu\mu}_{\mathbf{q},\mathbf{k}-\mathbf{q};\mathbf{k}}\big|^2$ in the decay rate (17) by a constant, thus eliminating the numerically complex and costly element of the calculation and bypassing its analytical cumbersomeness. As a result, the decay rate (17) is simply proportional to the on-shell two-magnon DOS (18)

$$\gamma^{\mu}_{\mathbf{k}} \approx \frac{f}{2}\big|\widetilde{J}^{\text{odd}}\big|^2 D^{\mu}_{\mathbf{k}}, \tag{19}$$

with $f = \langle\big|\widetilde{\Phi}^{\eta\nu\mu}_{\mathbf{q},\mathbf{k}-\mathbf{q};\mathbf{k}}\big|^2\rangle$ and brackets implying averaging. We argue that this approximation is well-justified for the gapped systems and for models with lower spin symmetries. This is because Bogolyubov transformations are not singular without the true Goldstone modes and because the lower symmetry implies fewer restrictions on the decay amplitudes beyond the kinematic constraints in the DOS, see [26] and [30].

Another strong à posteriori justification of this procedure comes from the need of a regularization of the imprints of the two-magnon Van Hove singularities in the Born decay rate $\gamma^{\mu}_{\mathbf{k}}$ (17) that are inherited from the two-magnon DOS (18). Such singularities are unavoidable, see [26], yet they are unphysical and must be regularized. Regularization procedures result in an averaging of singularities over the momentum space, thus complementing the averaging suggested in our approximation (19). We also note that performing such a regularization in case of the fully numerical calculation of the vertex $\widetilde{\Phi}^{\eta\nu\mu}_{\mathbf{q},\mathbf{k}-\mathbf{q};\mathbf{k}}$ can be prohibitively costly.

The method that we use to address the issue of singularities is referred to as the iDE method, e.g. [31, 32]. It is a simplified version of Dyson's equation on the pole with only imaginary part of the equation solved self-consistently, which is in line with the approximations described above, $\Sigma_{\mu\mathbf{k}}(\varepsilon_{\mu\mathbf{k}} + i\gamma^{\mu}_{\mathbf{k}}) = -i\gamma^{\mu}_{\mathbf{k}}$. Allowing the initial magnon to have a finite lifetime relaxes the energy and momentum conservations, thus removing singularities of the Born approximation and mitigating the unphysical largeness of the $\gamma^{\mu}_{\mathbf{k}}$'s. Technically, instead of the integral in (17) with a simplifying assumption of (19), the calculation of $\gamma^{\mu}_{\mathbf{k}}$ now requires a recursive solution of

$$1 = \frac{f}{2}\sum_{\mathbf{q},\nu\eta} \frac{\big|\widetilde{J}^{\text{odd}}\big|^2}{(\varepsilon_{\mu\mathbf{k}} - \varepsilon_{\nu\mathbf{q}} - \varepsilon_{\eta\mathbf{k}-\mathbf{q}})^2 + (\gamma^{\mu}_{\mathbf{k}})^2}, \tag{20}$$

which is, typically, a quickly convergent process. To provide a reasonable estimate of the constant f, we use our previous study of the XXZ model on the same (honeycomb) lattice in external field [30], for which analytical expressions for the dimensionless cubic vertices $\widetilde{\Phi}^{\eta\nu\mu}_{\mathbf{q},\mathbf{k}-\mathbf{q};\mathbf{k}}$ can be obtained. In this model [30], the characteristic values of the three-magnon couplings $\widetilde{J}^{\text{odd}}$ relative to the magnon bandwidth are close to the ones considered in the current study, single-magnon states significantly overlap with the high-intensity parts of the two-magnon continuum in high fields, also in a close similarity to the kinematics of the present work, and magnon decays were shown to be very significant. By comparing Born-approximation $\gamma^{\mu}_{\mathbf{k}}$ for the XXZ model with the corresponding two-magnon density of states [30], we extract the value of $f \simeq 1/9$. Back-of-the-envelope estimates suggest this constant to be $f \simeq 4/zn^2$, where z is the coordination number and n is the number of magnon branches (sites in the unit cell). This estimate comes from analyzing the structure of the dimensionless cubic vertices $\widetilde{\Phi}^{\eta\nu\mu}_{\mathbf{q},\mathbf{k}-\mathbf{q};\mathbf{k}}$ in previous studies such as Supplementary Ref. [30]. As is clear from Supplementary Eq. (14), the real-space coupling of spin-flips affects nearest-neighbour Holstein-Primakoff magnons. Hence, the vertex in **k**-space contains an

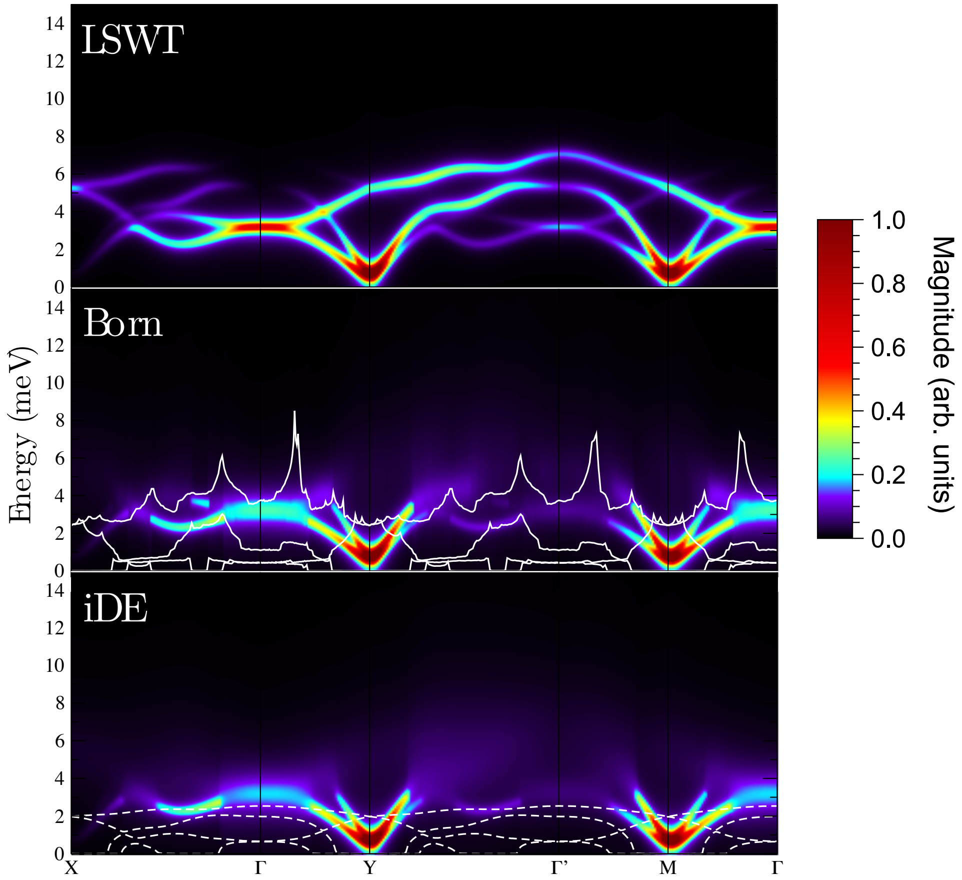

SUPPLEMENTARY FIGURE 4. **Comparison of LSWT, Born and iDE.** The ordering vector in the $\mathcal{I}(\mathbf{q},\omega)$ intensity plots was chosen to be at the M′-point $[(-\pi,\pi/\sqrt{3})]$. Upper panel: Linear spin wave theory (LSWT) along the contour XΓYΓ′MΓ in Fig. 3(b) of the main text with artificial broadening $\delta = 0.25$ meV. Middle panel: same with broadening $\gamma^{\mu}_{\mathbf{q}}$ from Supplementary Eq. (19) representing the approximate Born expression. Lower panel: same with broadening $\gamma^{\mu}_{\mathbf{q}}$ from Supplementary Eq. (20) representing the self-consistent imaginary Dyson equation (iDE) approach. Solid and dashed lines are $\gamma^{\mu}_{\mathbf{q}}$ from (19) and (20), respectively, for the four magnon modes with the higher values corresponding to the higher-energy modes.

analog of the nearest-neigbour hopping matrix. Averaging of its square yields with $\sim 1/z$ the inverse coordination number. The number of atoms in the magnetic unit cell n gives the number of independent magnon modes and, therefore, their wavefunctions are normalized by $1/\sqrt{n}$. Since the vertex couples three magnons, its square is, thus, proportional to $\sim 1/n^3$. The summation over such modes eliminates one power of n. The factor of 4 comes from the square of the symmetrization factor in the decay term. For the considered problem of $n = 4$ and $z = 3$ the value of $f \simeq 1/12$ is in a quantitatively close agreement with the value of $f \simeq 1/9$.

Dynamical Structure Factor

Our results for the dynamical structure factor $\mathcal{I}(\mathbf{q},\omega)$ for Model 2 are presented in Supplementary Figs. 4 and 5. Supplementary Figure 4 shows $\mathcal{I}(\mathbf{q},\omega)$ for the zigzag state with

the ordering vector at the M′-point $[(-\pi,\pi/\sqrt{3})]$, with that choice motivated by a close similarity of $\mathcal{I}(\mathbf{q},\omega)$ along the XKΓYΓ′MΓ **k**-path (see Fig. 3(c) of the main text) with the one averaged over three zigzag configurations, shown in Supplementary Figure 5.

The upper panels in both Supplementary Figures show the LSWT results with an artificial Lorentzian broadening of $\delta = 0.25$ meV. The middle and the lower panels in Supplementary Figure 4 and the two lower panels in Supplementary Figure 5 show $\mathcal{I}(\mathbf{q},\omega)$ with the broadened magnon lines according to

$$\delta(\omega-\varepsilon_{\mu\mathbf{q}}) \to A_\mu(\mathbf{q},\omega) = \frac{1}{\pi}\frac{\gamma^\mu_\mathbf{q}}{(\omega-\varepsilon_{\mu\mathbf{q}})^2+(\gamma^\mu_\mathbf{k})^2}, \tag{21}$$

where the broadening is obtained from the approximate Born expression (19) for the middle panel of Supplementary Figure 4 and by the iDE method (20) described above for the rest of the panels. The second panel in Supplementary Figure 5 shows exact diagonalization results from Fig. 3(a) of the main text.

The solid lines in the middle panel of Supplementary Figure 4 show Born approximation decay rates $\gamma^\mu_\mathbf{q}$ from (18) with the three-magnon coupling $\widetilde{J}^{\text{odd}}$ from (16) and $f = 1/9$ as discussed above. For each **k**-point, there are four values of $\gamma^\mu_\mathbf{q}$, one for each branch, with the larger value corresponding to the magnon that is higher in energy. With $\gamma^\mu_\mathbf{q}$ reaching about a half of the total magnon bandwidth, there is hardly anything visible left from the intensity of the highest-energy mode. The second highest mode is also overdamped in most of the **k**-space. This is in agreement with the high-energy magnons having a significant two-magnon continuum phase space for decays.

The two lower-energy modes are also broadened, with the modulations of the broadening following the **k**-regions where magnons do or do not overlap with the two-magnon continuum, such as in the vicinity of the Y and M points in Supplementary Figs. 4 and 5 for the latter case.

Last but not the least are the Van Hove singularities in $\gamma^\mu_\mathbf{q}$ from the two-magnon DOS. Their imprints are also visible in $\mathcal{I}(\mathbf{q},\omega)$ as sharp boundaries between more or less bright regions of intensity, hence more or less well-defined magnons. At **k**'s close to such singularities, the decay rates become unphysically large, violate the perturbative nature of the expansion, and need to be regularized.

Physically, since the Van Hove singularities are affecting magnons that are already within the two-magnon continuum and are, thus, decaying, the most relevant method for such a regularization is the iDE approach described above, which allows to self-consistently account for the broadening of the initial-state magnons.

The iDE broadening $\gamma^\mu_\mathbf{q}$ from (20) is shown in the lower panel of Supplementary Figure 4 by the dashed lines. As one can see, the iDE method yields smooth, completely regular decay rates, with their overall values decreased for the upper- and somewhat increased for the lower-energy modes. We emphasize again that this result is more realistic than that of the Born approximation, because the divergent behaviour violates the perturbative nature of the $1/S$ expansion and is unphysical.

Supplementary Figure 5 shows $\mathcal{I}(\mathbf{q},\omega)$ averaged over three zigzag configurations. Our iDE results in the first lower panel of Supplementary Figure 5 clearly capture many of the most notable features seen in the ED data and constitute a clear improvement over the LSWT results. There are still some notable differences. First, in the vicinities of the M- and Y-points, ED branches at lower energies are flatter and asymmetric with a more drastic decrease of intensity in the Y→ Γ′ direction. There is also only one mode resolved near

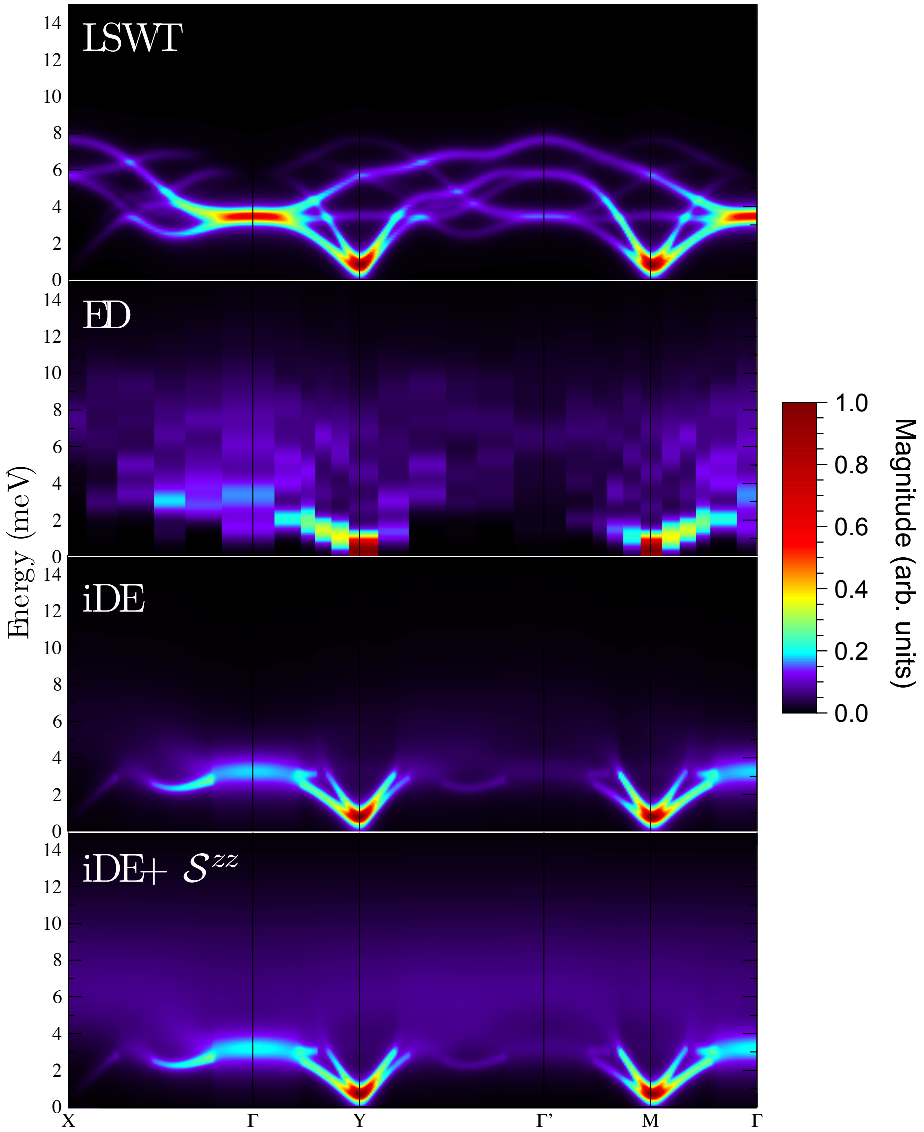

SUPPLEMENTARY FIGURE 5. **Averaged $\mathcal{I}(\mathbf{q},\omega)$ in various approaches.** $\mathcal{I}(\mathbf{q},\omega)$ intensity plots are averaged over the three zigzag states. Upper panel: LSWT with artificial broadening $\delta = 0.25$ meV. Second upper panel: ED results from Fig. 3(c) of the main text. First lower panel: LSWT with the iDE broadening $\gamma_{\mathbf{q}}^{\mu}$ from Supplementary Eq. (20). Second lower panel: same with the averaged longitudinal intensity $\mathcal{I}^{zz}(\mathbf{q},\omega)$, see text.

these points in the ED data, while the SWT predicts two. These may be ascribed to the ignoring of the real part of the self-energies, which neglects the spectrum flattening and the reduction of the quasiparticle peak intensity.

Another major remaining difference is the presence of a significant intensity in the ED data at the energies near and above the single-magnon band maximum, $\omega \gtrsim 7$ meV, which is completely missing in both LSWT and iDE results. This missing feature is beyond standard calculations of the structure factor, which take into account only transverse spin fluctuations (see Supplementary Ref. [33] for an exception). The missing part is the longitudinal component, $\mathcal{I}^{zz}(\mathbf{q},\omega)$ in the local z-axes, which is directly related to the continuum of the

broadened two-magnon excitations. While the full calculation of $\mathcal{I}^{zz}(\mathbf{q},\omega)$ is beyond the scope of the current work, a simple account of its ω structure is possible.

A naïve approach is to suggest a direct proportionality of $\mathcal{I}^{zz}(\mathbf{q},\omega)$ to the two-magnon DOS, similarly to Supplementary Eq. (19). However, a better approximation is achieved by modifying the density of states by including the broadenings of the magnon lines inside the continuum

$$\mathcal{I}^{zz}(\mathbf{q},\omega) = \frac{f_2}{2} \sum_{\mathbf{k},\mu\nu} \frac{\gamma^{\mu}_{\mathbf{k}} + \gamma^{\nu}_{\mathbf{k}-\mathbf{q}}}{(\omega - \varepsilon_{\mu\mathbf{k}} - \varepsilon_{\nu\mathbf{k}-\mathbf{q}})^2 + (\gamma^{\mu}_{\mathbf{k}} + \gamma^{\nu}_{\mathbf{k}-\mathbf{q}})^2}. \tag{22}$$

This modification is very physical and self-consistent as the continuum is built from the broadened magnons. It also eliminates sharp features in the continuum and because the $\gamma^{\mu}_{\mathbf{q}}$'s are larger for the upper magnon branches, the upper part of the continuum also gets washed out more. The single adjustable parameter f_2 can be argued to be 1/8 using naïve estimates. While the resulting intensity lacks a more involved modulation in the momentum, one can expect an overall better description of the ED results.

The results shown in the lowest panel of Supplementary Figure 5 include $\mathcal{I}^{zz}(\mathbf{q},\omega)$ from (22) with $f_2 = 1/8$ and the averaged iDE decay rates: $\gamma^1_{\mathbf{q}} = 0.5$ meV, $\gamma^2_{\mathbf{q}} = 1.0$ meV, $\gamma^3_{\mathbf{q}} = 1.5$ meV, and $\gamma^4_{\mathbf{q}} = 2.5$ meV, where the numeration is from the lowest to the highest in energy.

Thus, the broad features in the full $\mathcal{I}(\mathbf{q},\omega)$ are a combination of the remnants of the broadened magnon modes from the transverse part of $\mathcal{I}(\mathbf{q},\omega)$ with the longitudinal $\mathcal{I}^{zz}(\mathbf{q},\omega)$ part, so the combination has a maximum at the energies between 6 and 7 meV, in a close resemblance of the ED data. While this is only an approximate description, it provides confidence that a complete account should be able to reproduce other features of the ED data in that range of energies as well.

Altogether, we believe we have been able to provide a convincing description of the most significant effects of the three-magnon interaction on the magnon spectrum that agrees with the numerical studies.

SUPPLEMENTARY NOTE 4: FURTHER DETAILS OF EXACT DIAGONALIZATION CALCULATIONS

The exact diagonalization calculations in this work were carried out on the series of 20- and 24-site clusters with periodic boundary conditions shown in Supplementary Figure 6. As noted in the Methods section of the main text, ED calculations were performed using the Lanczos algorithm [34], employing the continued fraction method [35] to obtain the desired dynamical correlation functions. While the periodic cluster **24A** retains all the symmetries of Supplementary Eq. (1), the remaining clusters are of lower symmetry, resulting in slight anomalies in the symmetries of the computed correlation functions. For **24B**, **20A**, and **20B** the symmetry was partially restored by averaging the results over all symmetry-related orientations of the clusters, which generates the **k**-points shown in Supplementary Figure 6(b).

In the following pages, we show complete results obtained for various models. For each model, comparison of results for each cluster is shown for the high-symmetry Γ, X, M(Y) and Γ′ points, which live on all four clusters. These results appear in Supplementary Figure 8-10(d,h,l,p). For each case, the lowest energy peak positions are relatively well converged with respect to finite-size effects (compared to the chosen 0.5 meV Gaussian broadening). For

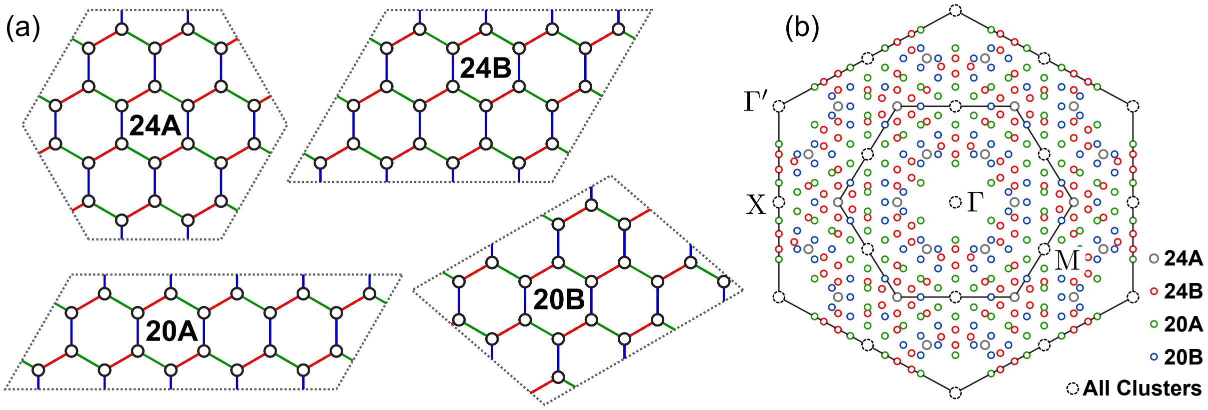

SUPPLEMENTARY FIGURE 6. **Cluster details in real and reciprocal space.** (a) 20- and 24- site periodic clusters employed for exact diagonalization (ED) studies in this work. (b) Summary of **k**-points associated with each cluster. For the low-symmetry **20A**, **20B**, and **24B**, **k**-points are shown for all symmetry-related orientations of the cluster. The high-symmetry points Γ, X, M(Y) and Γ′ live on all clusters.

excitations representing a continuum, we observe variations in the positions of the higher energy peaks obtained from the various clusters, as might be expected. Averaging over the discrete excitations of the different clusters therefore restores the continuum, improving the validity of the computed intensities at the high-symmetry Γ, X, M(Y) and Γ′ points. However, we note that away from the high-symmetry points, where averaging is not possible, the intensities are less reliable. This observation does not alter the conclusions drawn from plotting $\mathcal{I}(\mathbf{k},\omega)$ along various **k**-paths (as in Supplementary Figure 7), but should be noted. In particular, in order to avoid spurious features, the plots of the **k**-dependence of $\mathcal{I}(\mathbf{k},\omega)$ for various energy intervals (Fig. 2(d) and 3(d), main text, and Supplementary Figure 8-10(a,e,i,m)) employed only data from the highest symmetry **24A** cluster. Plots of $\mathcal{I}(\mathbf{k},\omega)$ along the particular **k**-path (Fig. 2(c) and 3(c), main text, and Supplementary Figure 8-10(b,f,j,n)) employed data from all clusters. In Supplementary Figure 7 we identify the periodic cluster associated with each **k**-point.

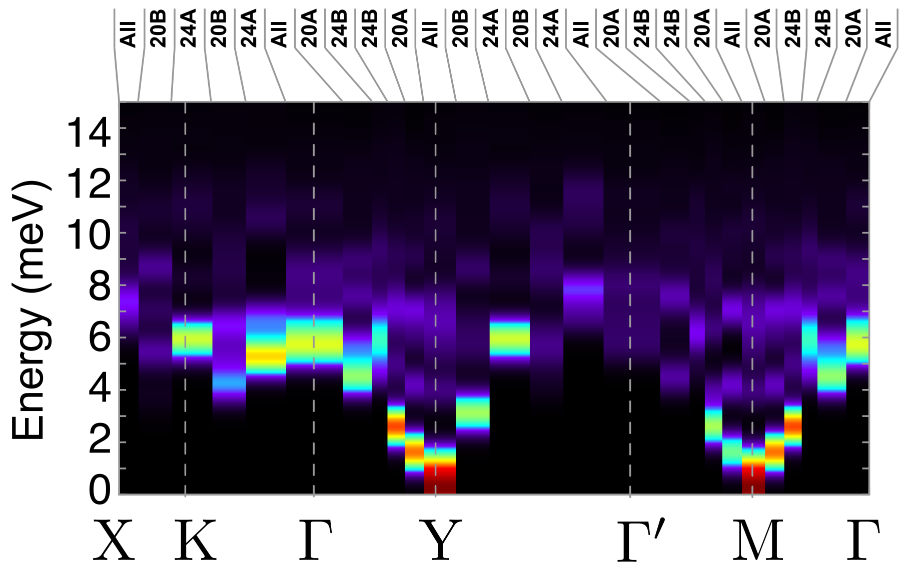

SUPPLEMENTARY FIGURE 7. **Specific contributions of periodic clusters.** Predicted $\mathcal{I}(\mathbf{k}, \omega)$ for $J_1 = -4.6, K_1 = +7.0$ meV showing the periodic cluster of origin for each momentum interval. For intervals labelled "All" the obtained intensities were averaged over the four clusters. Comparison of the intensities at such **k**-points is shown in Supplementary Figure 8-10(d,h,l,p).

SUPPLEMENTARY NOTE 5: ADDITIONAL ED RESULTS FOR VARIOUS MODELS

In this section, we show full results for various additional parameters not appearing in the main text, as well as a comparison of results from the different periodic clusters **20A-24B**.

Nearest Neighbour Heisenberg-Kitaev (nnHK) Model

We first show, in Supplementary Figure 8, results obtained for the nnHK model with $K_1 > 0$ and $J_1 < 0$. We begin with $J_1 = -4.6$ meV, $K_1 = +7.0$ meV (Supplementary Figure 8(a-d)), as suggested from analysis of powder inelastic neutron scattering data in Supplementary Ref. [25], and then consider several models moving towards the spin-liquid, maintaining constant $\sqrt{K_1^2 + J_1^2}$.

The validity of the ED approach can be seen by comparing the results for the pure Kitaev model (Supplementary Figure 8(n)) with the exact results (Supplementary Figure 8(o)). One can see good agreement between $\mathcal{I}(\mathbf{k}, \omega)$ predicted from the two approaches. A similar degree of agreement is seen for $J_1 = -4.6$ meV, $K_1 = +7.0$ meV, for which the ED results (Supplementary Figure 8(b)) and the LSWT results (Supplementary Figure 8(c)) are also in close correspondence. This model exists close to the hidden SU(2) point (at $K_1 = -2J_1$) noted in Supplementary Ref. [7]. For this reason, the dynamics are expected to be well described by conventional spin-waves. Therefore, the LSWT method performs well both close to and far away from the spin liquid.

As discussed in the main text, upon approaching the spin-liquid, the intensive excitations at the 2D Γ-point shift to higher energy, and become increasingly broad as they move deeper into the three-magnon continuum. Away from the Γ-point, the intensive excitations are mostly associated with the lowest magnon band at the level of LSWT. These excitations remain relatively sharp in the ED calculations, and shift to lower energy on approaching the spin-liquid. This effect is clearly seen for the computed intensity at the X-point, shown in Supplementary Figure 8(d,h,l,p), which remains sharply peaked over the entire studied range. As discussed in the main text and Supplementary Note 3, low-energy magnons in the nnHK model are protected from decay due to the absence of three-magnon states at low energies. As shown in Supplementary Figure 8(n,o), these low energy excitations eventually evolve into a flat band in the spin liquid with intensity peaked just above the two-flux gap $\Delta \sim 0.065|K_1|$ [12, 13].

As in Supplementary Ref. [36], we find that the best agreement with the experimental results within the nnHK model is obtained for $|J_1/K_1| = 0.3$, which shows a star-like pattern at intermediate energy. This ratio of interactions was featured in the main text, for Model 1. However, we also find significant intensity at the X-points, inconsistent with the observed intensities. Moreover, as noted in the main text, the absence of low-energy intensity at the Γ-point in these models is strongly inconsistent with the experimental data of Supplementary Refs. [17, 19].

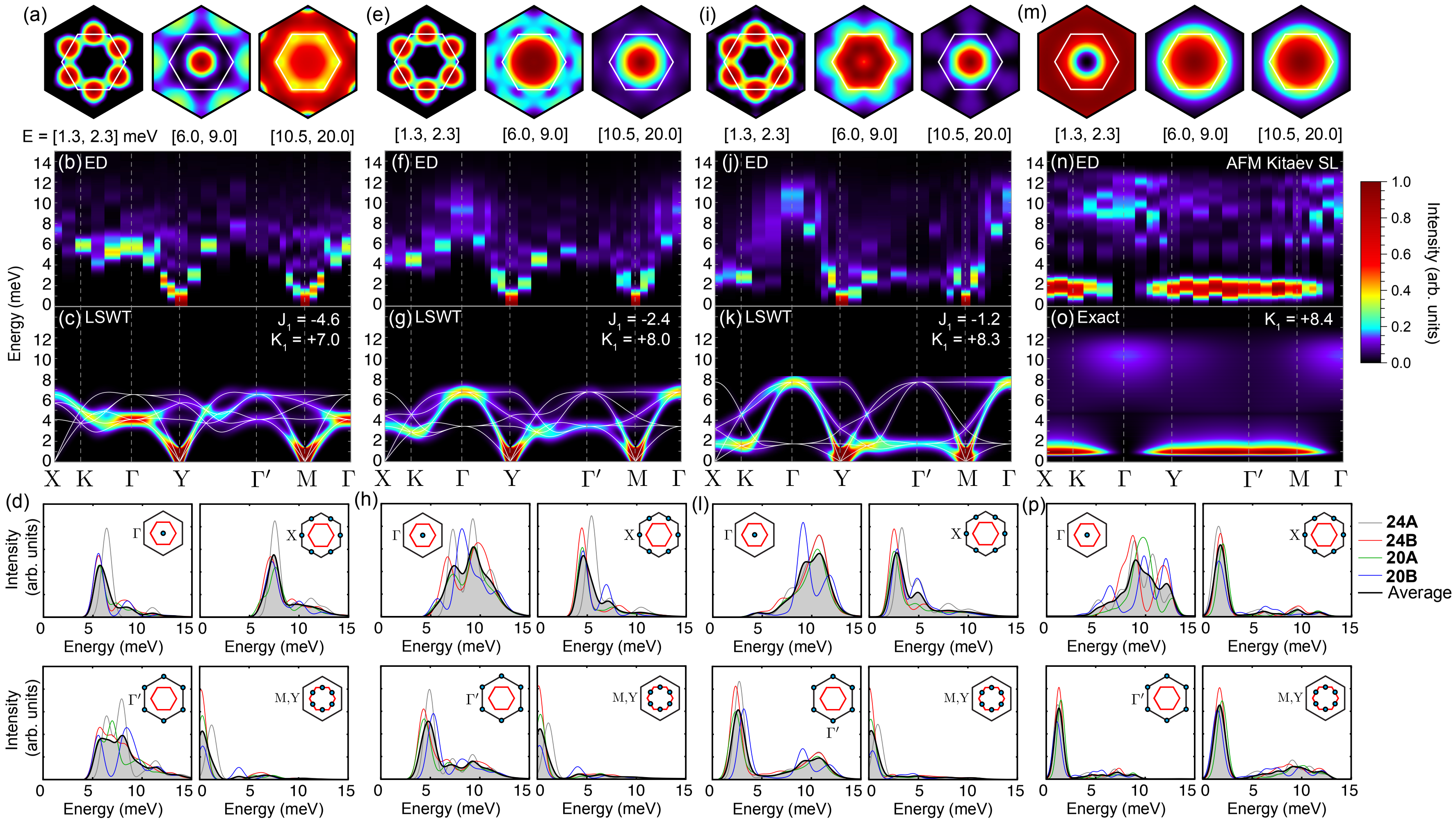

SUPPLEMENTARY FIGURE 8. **Detailed results for the nnHK model.** For each model we show, for example, (a) **k**-dependence of intensity integrated over the indicated energy regions, (b) ED and (c) LSWT intensities over the indicated **k**-path. (d) Comparison of the intensities obtained from the various clusters; intensities are not shown to scale between different **k**-points. Analogous results are shown in (e)-(p). For the spin-liquid, (m) and (o) represent exact results (see Supplementary Refs. [12, 13]).

Extended $(J_1, K_1, \Gamma_1, J_3)$ Model, $K_1 > 0$

We next consider parameters in the region suggested by *ab initio* studies of the earlier $P3_112$ structure of α-$RuCl_3$: $|J_1| \sim |K_1| \sim |\Gamma_1|$, with $J_1 < 0, K_1 > 0, \Gamma_1 > 0$ [8, 21]. It is worth noting that this region of the phase diagram features competition between ferromagnetic, zigzag, and 120° order, such that the combination of $K_1 > 0$ and a finite $\Gamma_1 > 0$ tends to destabilize the zigzag order. In order to restore the zigzag ground state, we therefore add a small J_3 coupling. We start with the interactions suggested in Supplementary Ref. [25], namely $J_1 = -4.6$ meV, $K_1 = +7.0$ meV. We then add a small $J_3 = 0.7$ meV, and then vary the ratio of $|\Gamma_1/K_1|$, holding constant J_1, J_3, and $\sqrt{J_1^2 + K_1^2 + \Gamma_1^2 + J_3^2}$. Results are shown in Supplementary Figure 9.

Comparing Supplementary Figure 9(a-d) with Supplementary Figure 9(e-h), one can see that the addition of a small J_3 does not significantly influence the spectra. However, similar to the $K_1 < 0$ region, we observe significant broadening of the ED spectra upon increasing Γ_1. For $(J_1, K_1, \Gamma_1, J_3) = (-4.6, +5.0, +5.0, +0.7)$ meV (Supplementary Figure 9(m-p)), LSWT predicts a large spin-wave gap, and relatively flat dispersion for the spin-wave bands. Interestingly, the momentum dependence of the predicted intensities from LSWT resemble somewhat those of the $K_1 < 0$ Kitaev spin-liquid, but shifted to higher energy. That is, there appears a flat band at 4-5 meV, with intensity centered around the 2D Γ-point, and another band at higher energies 10-12 meV, with intensity away from the Γ-point. The vanishing dispersion of these bands at the level of LSWT is likely related to close proximity to the phase boundaries between ferromagnetic, zigzag, and 120° order, which would typically feature low-energy modes near the Γ, M(Y), and K-points, respectively.

Results for the ED calculations differ significantly from the LSWT intensities at large Γ_1. In particular, the gap is significantly reduced, such that dispersing modes can be observed near the (M,Y)-points. This may result from shifting of the phase boundaries in the ED calculations compared to the semiclassical LSWT approach. Interestingly, for $(J_1, K_1, \Gamma_1, J_3) = (-4.6, +5.0, +5.0, +0.7)$ meV (Supplementary Figure 9(m-p)) we observe low-energy intensity at the Γ-point in the ED calculations, and a star-like shape at intermediate energies, consistent with the experimental data on α-$RuCl_3$. However, in the high energy region, the intensity is mainly located away from the Γ-point, in contradiction with the experiment of Supplementary Ref. [19].

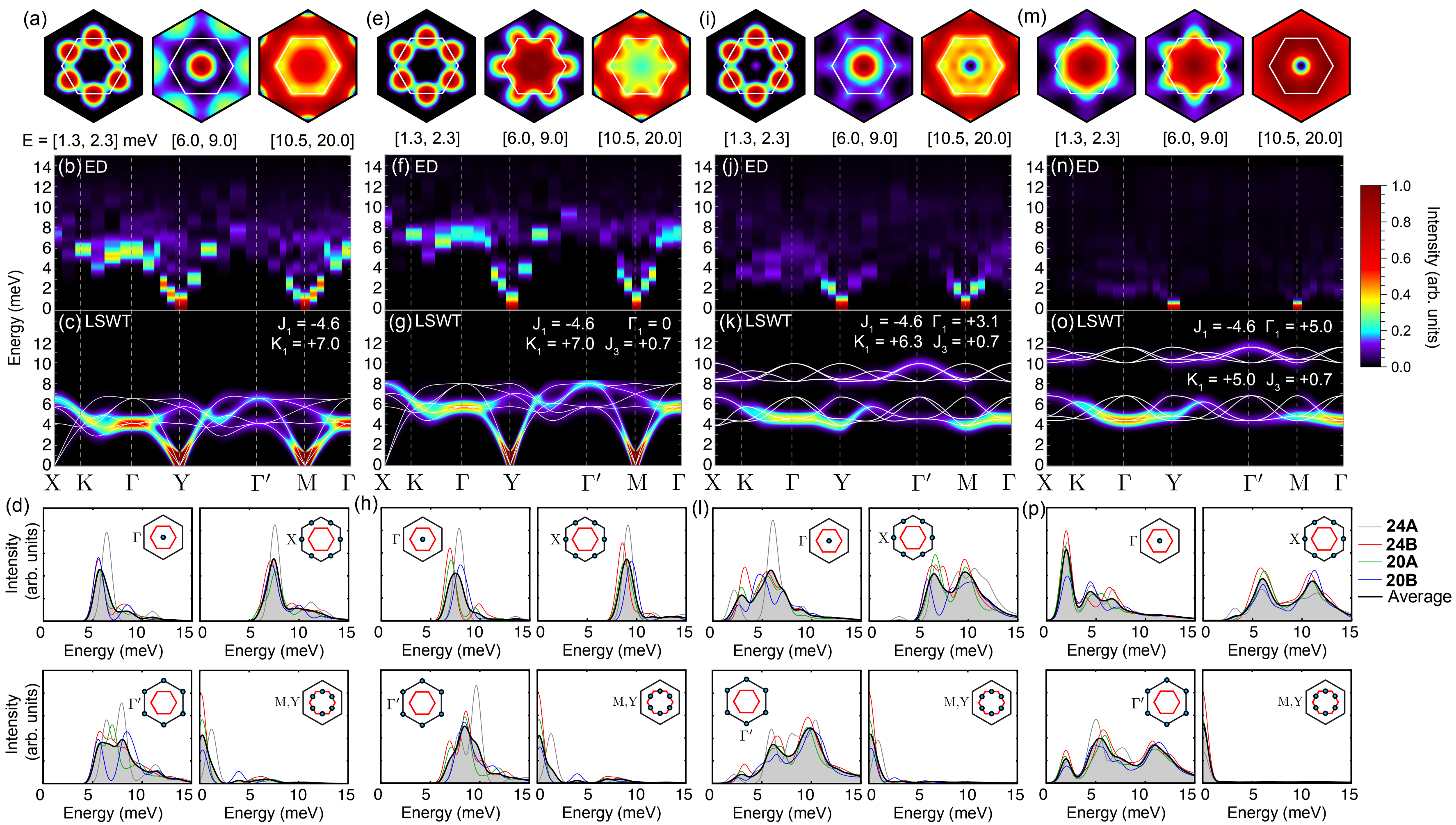

SUPPLEMENTARY FIGURE 9. **Detailed results for the extended model with $K_1 > 0$.** For each model we show, for example, (a) **k**-dependence of intensity integrated over the indicated energy regions, (b) ED and (c) LSWT intensities over the indicated **k**-path. (d) Comparison of the intensities obtained from the various clusters; intensities are not shown to scale between different **k**-points. Analogous results are shown in (e)-(p).

Extended $(J_1, K_1, \Gamma_1, J_3)$ Model, $K_1 < 0$

Finally, we show, in Supplementary Figure 10, complete results for a variety of models in the region suggested by various *ab initio* studies based on recent $C2/m$ structures of α-$RuCl_3$, that is $J_1 \sim 0, K_1 < 0, \Gamma_1 > 0, J_3 > 0$ [8, 10, 22–24]. In each case, we hold $J_1 = -0.5$ meV, $J_3 = +0.5$ meV and the overall scale $\sqrt{J_1^2 + K_1^2 + \Gamma_1^2 + J_3^2}$ constant, and modify the ratio of $|K_1/\Gamma_1|$. We also show results for the $K_1 < 0$ Kitaev spin-liquid for comparison.

As discussed in the main text and Supplementary Note 3, a large Γ_1 interaction induces significant deviations between the ED and LSWT results, due to coupling between the one- and two-magnon excitations. For Supplementary Figure 10(b,f), only the excitations around the (M,Y)-points are sharply peaked in the ED calculations. On approaching the spin-liquid (by decreasing $|\Gamma_1/K_1|$), the continuum is shifted to lower energies, and becomes sharper, evolving into the flat sharp band in the spin-liquid. The importance of Γ_1 interactions can further be seen by comparing the results of Supplementary Figure 8(a-d) with Supplementary Figure 10(a-d). For these models, the LSWT predictions for $\mathcal{I}(\mathbf{k}, \omega)$ are very similar despite remarkably different interaction parameters. However, the ED results differ substantially.

As noted in the main text and Supplementary Note 3, the requirements for strong coupling of the one- and two-magnon excitations include a deviation of the ordered moments from the high symmetry cubic axes. While finite Γ_1 interactions generally rotate the ordered moments away from the cubic axes, it is interesting to consider also the case where $\Gamma_1 = 0$, $J_3 > 0$, and $K_1 < 0$. In this case, the directions of the ordered moments are not completely determined at the classical level. For example, for the ordering wavevector $\mathbf{Q}$ parallel to the Z bond, the classical energy is minimized for any orientation of the moments in the xy-plane. However, as noted in Supplementary Refs. [27, 37] the cubic axes are selected by a quantum order-by-disorder mechanism, such that magnons are expected to remain stable in this limit. Indeed, comparison of the ED and LSWT results in Supplementary Figure 10(j) with (k) shows general agreement. This observation further establishes the importance of off-diagonal anisotropic interactions such as Γ_1.

Finally, we note that the models studied in this region (such as Model 2 of the main text) display excitation gaps on the order of 0.5 meV (at the M(Y)-points) at the level of both ED and LSWT. This is in contrast with the neutron scattering results, which appear to show a gap on the order of 2 meV [25, 38]. However, it is worth noting that the size of the excitation gap is strongly influenced by the relative magnitudes of the K_1 and Γ_1 interactions along each nearest neighbour X,Y and Z bond, which are not constrained to be equal by the symmetry of the real crystals [8, 10]. To demonstrate this, we show LSWT results for Model 2 (Supplementary Figure 11), modified with an anisotropic K_1 and Γ_1, consistent with the results of Supplementary Ref. [8]. Specifically, we show $J_1 = -0.5$, $J_3 = +0.5$, with $K_1^Z = -5.0 + \delta, K_1^{XY} = -5.0 - \delta, \Gamma_1^Z = +2.5 + \delta/2, \Gamma^{XY} = +2.5 - \delta/2$. The gap can be reproduced already for small perturbations on the order of $\delta = 0.1K_1$, while the remainder of the dispersions are not strongly affected. Furthermore, additional small anisotropic interactions [39–41] are allowed by symmetry that may also contribute to the gap. For simplicity, we have neglected such additional terms in the main text, but expect that their inclusion would further improve agreement with the experimental observations.

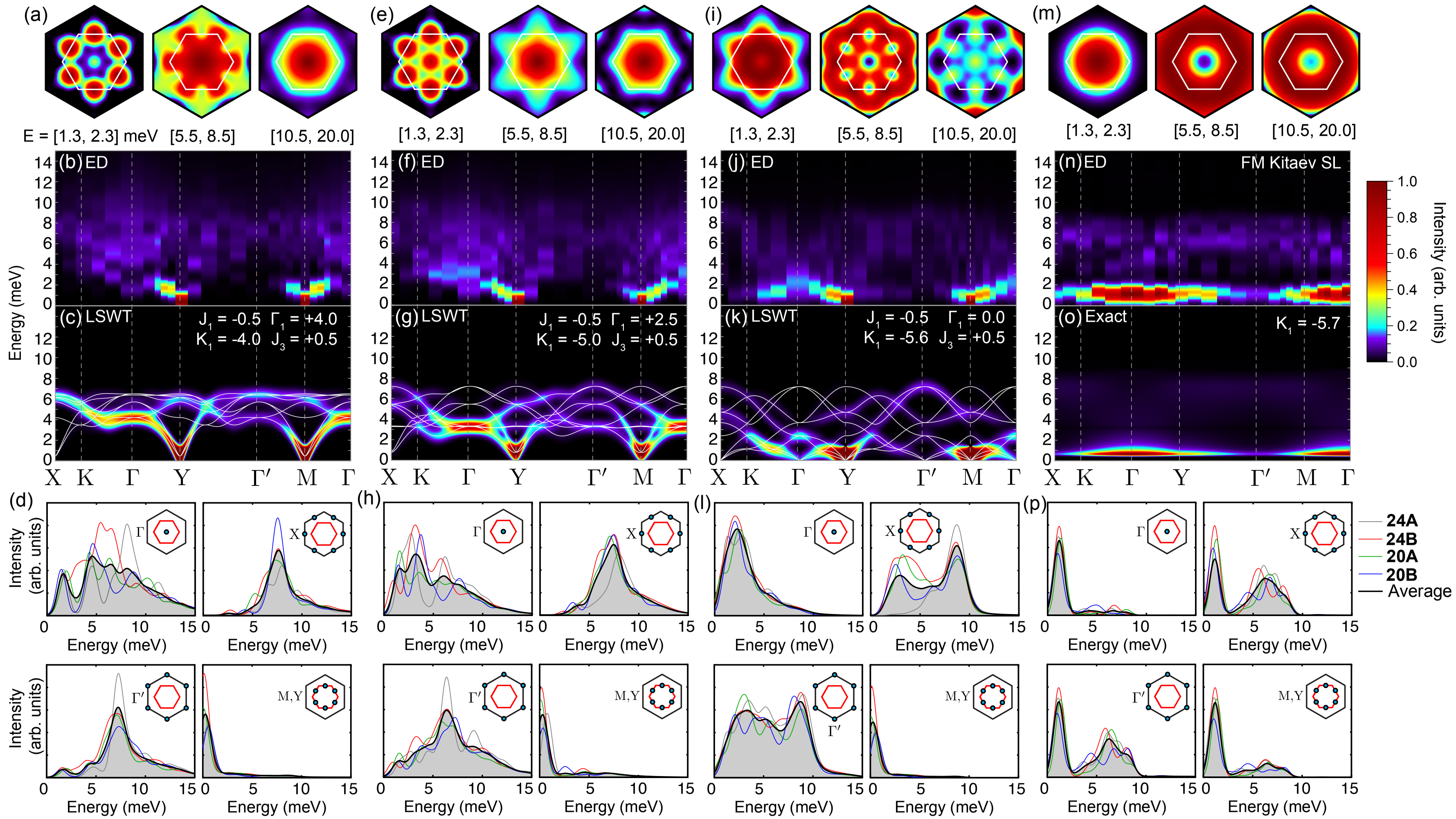

SUPPLEMENTARY FIGURE 10. **Detailed results for the extended model with $K_1 < 0$.** For each model we show, for example, (a) **k**-dependence of intensity integrated over the indicated energy regions, (b) ED and (c) LSWT intensities over the indicated **k**-path. (d) Comparison of the intensities obtained from the various clusters; intensities are not shown to scale between different **k**-points. Analogous results are shown in (e)-(p). For the spin-liquid, (m) and (o) represent exact results (see Supplementary Refs. [12, 13]).

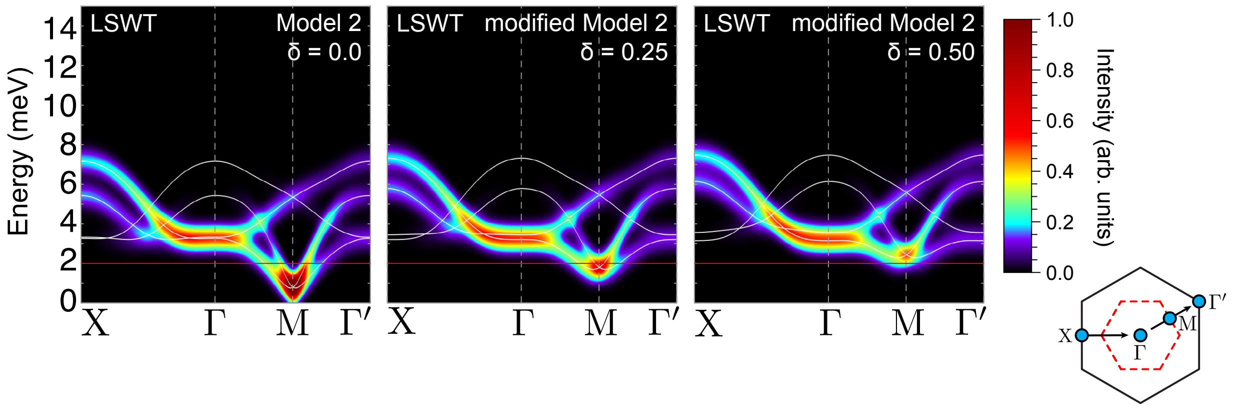

SUPPLEMENTARY FIGURE 11. **Bond-dependent interactions: Evolution of the gap.** Bond-dependent interactions are introduced at the LSWT level to investigate the evolution of the gap at the M-point. The ordering wavevector is the Y-point in each case, and results are not averaged over 120 degree domains. The experimental value of ~ 2.0 meV is indicated by a red line. Relatively small perturbations are sufficient to reproduce the experimental gap.

SUPPLEMENTARY REFERENCES

[1] Gotfryd, D. *et al.* Phase diagram and spin correlations of the Kitaev-Heisenberg model: Importance of quantum effects. *Phys. Rev. B* **95**, 024426 (2017).

[2] Chaloupka, J., Jackeli, G. & Khaliullin, G. Kitaev-Heisenberg Model on a Honeycomb Lattice: Possible Exotic Phases in Iridium Oxides A_2IrO_3. *Phys. Rev. Lett.* **105**, 027204 (2010).

[3] Baskaran, G., Mandal, S. & Shankar, R. Exact Results for Spin Dynamics and Fractionalization in the Kitaev Model. *Phys. Rev. Lett.* **98**, 247201 (2007).

[4] Rau, J. G., Lee, E. K.-H. & Kee, H.-Y. Generic Spin Model for the Honeycomb Iridates beyond the Kitaev Limit. *Phys. Rev. Lett.* **112**, 077204 (2014).

[5] Reuther, J., Thomale, R. & Trebst, S. Finite-temperature phase diagram of the Heisenberg-Kitaev model. *Phys. Rev. B* **84**, 100406 (2011).

[6] Schaffer, R., Bhattacharjee, S. & Kim, Y.-B. Quantum phase transition in Heisenberg-Kitaev model. *Phys. Rev. B* **86**, 224417 (2012).

[7] Chaloupka, J., Jackeli, G. & Khaliullin, G. Zigzag Magnetic Order in the Iridium Oxide Na_2IrO_3. *Phys. Rev. Lett.* **110**, 097204 (2013).

[8] Winter, S. M., Li, Y., Jeschke, H. O. & Valentí, R. Challenges in design of Kitaev materials: Magnetic interactions from competing energy scales. *Phys. Rev. B* **93**, 214431 (2016).

[9] Katukuri, V. M. *et al.* Kitaev interactions between $j = 1/2$ moments in honeycomb Na_2IrO_3 are large and ferromagnetic: insights from *ab initio* quantum chemistry calculations. *New J. Phys.* **16**, 013056 (2014).

[10] Yadav, R. *et al.* Kitaev exchange and field-induced quantum spin-liquid states in honeycomb α-$RuCl_3$. *Sci. Rep.* **6**, 37925 (2016).

[11] Kimchi, I. & You, Y.-Z. Kitaev-Heisenberg-J_2-J_3 model for the iridates A_2IrO_3. *Phys. Rev. B* **84**, 180407 (2011).

[12] Knolle, J., Kovrizhin, D. L., Chalker, J. T. & Moessner, R. Dynamics of a Two-Dimensional Quantum Spin Liquid: Signatures of Emergent Majorana Fermions and Fluxes. *Phys. Rev.*

Lett. **112**, 207203 (2014).

[13] Knolle, J., Kovrizhin, D. L., Chalker, J. T. & Moessner, R. Dynamics of fractionalization in quantum spin liquids. *Phys. Rev. B* **92**, 115127 (2015).

[14] Stroganov, E. V. & Ovchinnikov, K. V. Crystal structure of ruthenium trichloride. *Vestn. Leningr. Univ. Fiz. Khim* **12**, 152–157 (1957).

[15] Fletcher, J. M., Gardner, W. E., Fox, A. C. & Topping, G. X-Ray, infrared, and magnetic studies of α- and β-ruthenium trichloride. *J. Chem. Soc. A* 1038–1045 (1967).

[16] Johnson, R. D. *et al.* Monoclinic crystal structure of α-$RuCl_3$ and the zigzag antiferromagnetic ground state. *Phys. Rev. B* **92**, 235119 (2015).

[17] Cao, H. B. *et al.* Low-temperature crystal and magnetic structure of α-$RuCl_3$. *Phys. Rev. B* **93**, 134423 (2016).

[18] Kubota, Y., Tanaka, H., Ono, T., Narumi, Y. & Kindo, K. Successive magnetic phase transitions in α-$RuCl_3$: XY-like frustrated magnet on the honeycomb lattice. *Phys. Rev. B* **91**, 094422 (2015).

[19] Banerjee, A. *et al.* Neutron scattering in the proximate quantum spin liquid α-$RuCl_3$. *Science* **356**, 1055–1059 (2017).

[20] Morosin, B. & Narath, A. X-Ray Diffraction and Nuclear Quadrupole Resonance Studies of Chromium Trichloride. *J. Chem. Phys.* **40**, 1958–1967 (1964).

[21] Kim, H.-S., Shankar, V. V., Catuneanu, A. & Kee, H.-Y. Kitaev magnetism in honeycomb $RuCl_3$ with intermediate spin-orbit coupling. *Phys. Rev. B* **91**, 241110(R) (2015).

[22] Kim, H.-S. & Kee, H.-Y. Crystal structure and magnetism in α-$RuCl_3$: An *ab initio* study. *Phys. Rev. B* **93**, 155143 (2016).

[23] Hou, Y. S., Xiang, H. J. & Gong, X. G. Unveiling Magnetic Interactions of Ruthenium Trichloride via Constraining Direction of Orbital moments: Potential Routes to Realize Quantum Spin Liquid. Preprint at https://arxiv.org/abs/1612.00761 (2016).

[24] Wang, W., Dong, Z.-Y., Yu, S.-L. & Li, J.-X. Theoretical investigation of the magnetic dynamics and superconducting pairing symmetry in α-$RuCl_3$. Preprint at https://arxiv.org/abs/1612.09515 (2016).

[25] Banerjee, A. *et al.* Proximate Kitaev quantum spin liquid behaviour in a honeycomb magnet. *Nat. Mater.* **15**, 733–740 (2016).

[26] Zhitomirsky, M. E. & Chernyshev, A. L. *Colloquium*: Spontaneous magnon decays. *Rev. Mod. Phys.* **85**, 219–242 (2013).

[27] Chaloupka, J. & Khaliullin, G. Magnetic anisotropy in the Kitaev model systems Na_2IrO_3 and $RuCl_3$. *Phys. Rev. B* **94**, 064435 (2016).

[28] Plumb, K. W. *et al.* Quasiparticle-continuum level repulsion in a quantum magnet. *Nat. Phys.* **12**, 224–229 (2016).

[29] Hwang, K. & Kim, Y. B. Theory of triplon dynamics in the quantum magnet $BiCu_2PO_6$. *Phys. Rev. B* **93**, 235130 (2016).

[30] Maksimov, P. A. & Chernyshev, A. L. Field-induced dynamical properties of the *XXZ* model on a honeycomb lattice. *Phys. Rev. B* **93**, 014418 (2016).

[31] Chernyshev, A. L. & Zhitomirsky, M. E. Spin waves in a triangular lattice antiferromagnet: Decays, spectrum renormalization, and singularities. *Phys. Rev. B* **79**, 144416 (2009).

[32] Maksimov, P. A., Zhitomirsky, M. E. & Chernyshev, A. L. Field-induced decays in XXZ triangular-lattice antiferromagnets. *Phys. Rev. B* **94**, 140407(R) (2016).

[33] Mourigal, M., Fuhrman, W. T., Chernyshev, A. L. & Zhitomirsky, M. E. Dynamical structure factor of the triangular-lattice antiferromagnet. *Phys. Rev. B* **88**, 094407 (2013).

[34] Lanczos, C. An Iteration Method for the Solution of the Eigenvalue Problem of Linear Differential and Integral Operators. *J. Res. Nat. Bur. Stand.* **45**, 255–282 (1950).

[35] Dagotto, E. Correlated electrons in high-temperature superconductors. *Rev. Mod. Phys.* **66**, 763–840 (1994).

[36] Gohlke, M., Verresen, R., Moessner, R. & Pollmann, F. Dynamics of the Kitaev-Heisenberg Model. Preprint at https://arxiv.org/abs/1701.04678 (2017).

[37] Sizyuk, Y., Wölfle, P. & Perkins, N. B. Selection of direction of the ordered moments in Na_2IrO_3 and α-$RuCl_3$. *Phys. Rev. B* **94**, 085109 (2016).

[38] Ran, K. *et al.* Spin-Wave Excitations Evidencing the Kitaev Interaction in Single Crystalline α-$RuCl_3$. *Phys. Rev. Lett.* **118**, 107203 (2017).

[39] Sizyuk, Y., Price, C., Wölfle, P. & Perkins, N. B. Importance of anisotropic exchange interactions in honeycomb iridates: Minimal model for zigzag antiferromagnetic order in Na_2IrO_3. *Phys. Rev. B* **90**, 155126 (2014).

[40] Rousochatzakis, I., Reuther, J., Thomale, R., Rachel, S. & Perkins, N. B. Phase Diagram and Quantum Order by Disorder in the Kitaev $K_1 - K_2$ Honeycomb Magnet. *Phys. Rev. X* **5**, 041035 (2015).

[41] Rau, J. G. & Kee, H.-Y. Trigonal distortion in the honeycomb iridates: Proximity of zigzag and spiral phases in Na_2IrO_3. Preprint at https://arxiv.org/abs/1408.4811 (2014).
